# Iron deficiency and reduced exercise tolerance in adult Fontan patients

**DOI:** 10.3389/fped.2026.1766731

**Published:** 2026-03-11

**Authors:** Agnieszka Bartczak-Rutkowska, Olga Trojnarska, Sonia Nartowicz, Agata Markiewicz, Maciej Lesiak, Ewa Straburzyńska-Migaj

**Affiliations:** Cardiology Department, Poznan University of Medical Sciences, Poznan, Poland

**Keywords:** anaemia, congenital heart defect, exercise capacity, Fontan circulation, iron deficiency

## Abstract

**Background:**

The Fontan procedure allows patients with complex congenital heart defects unsuitable for biventricular repair to reach adulthood; however, it results in a non-physiological circulation characterized by chronically elevated systemic venous pressure, impaired preload, and multiple long-term complications. Iron deficiency (ID), a well-recognized determinant of reduced exercise capacity and adverse prognosis in heart failure, may also affect adults with Fontan circulation, yet data remain scarce. This study aimed to evaluate the prevalence, hematologic profile, and functional consequences of ID in adult Fontan patients.

**Methods:**

Twenty-seven adults after the Fontan procedure and 26 age- and sex-matched healthy controls were studied. Clinical data, hematologic indices, iron parameters (TSAT, ferritin, serum iron), and cardiopulmonary exercise test (CPET) variables were assessed. ID was defined as transferrin saturation (TSAT) < 20%.

**Results:**

ID was present in 21% of Fontan patients, exclusively among women. Compared with controls, Fontan patients had significantly reduced peak oxygen uptake and ventilatory efficiency. Iron-deficient Fontan patients demonstrated lower peak oxygen uptake, along with higher ventilatory efficiency slope and lower end-tidal carbon dioxide pressure. TSAT positively correlated with peak oxygen uptake, end-tidal carbon dioxide pressure, and negatively with ventilatory efficiency slope. Red cell distribution width showed inverse correlations with TSAT, and oxygen pulse.

**Conclusions:**

Some patients after the Fontan procedure exhibit a significant iron deficiency resulting from the hemodynamic consequences of this palliative circulation. This deficiency adversely affects their exercise capacity, underscoring the need for active monitoring of iron status parameters in this population.

## Introduction

The Fontan procedure represents one of the most groundbreaking achievements in cardiac surgery, enabling patients with complex congenital heart defects unsuitable for biventricular repair to reach adulthood. Separating the pulmonary and systemic circulations eliminates cyanosis, but it imposes a substantial hemodynamic burden on the single ventricle (1). In the absence of a subpulmonary ventricle, pulmonary blood flow becomes non-pulsatile and depends on both low pulmonary vascular resistance and, consequently, elevated systemic venous pressure. Although the Fontan procedure markedly improves survival, it is associated with numerous long-term complications, including heart failure, arrhythmias, thromboembolic events, hepatic congestion and frequent progression to cirrhosis, protein-losing enteropathy, and neurocognitive impairment (2, 3).

It has been demonstrated that in the general population, a substantial proportion of patients with heart failure (33%–74%) exhibit iron deficiency (ID), typically independent of the presence of anaemia (4, 5). ID is strongly associated with poorer prognosis, greater comorbidity, and increased mortality in these patients (6). The current definition of ID (ferritin <100 µg/L, or 100–299 µg/L with transferrin saturation <20%) was originally developed for patients with renal failure and does not fully reflect the mechanisms of ID in individuals with heart failure (7–10). A growing body of evidence indicates that transferrin saturation (TSAT) <20% is currently the most reliable and clinically practical criterion for identifying hypoferritinemia in the heart failure population (8).

It is also well established that reduced iron stores are found in patients with complex congenital heart defects, whose exercise capacity is typically impaired (11). An analysis of Fontan pathophysiology indicates several additional factors that may predispose these patients to ID. The absence of a subpulmonary ventricle leads to elevated venous pressure and, consequently, to hepatic congestion as well as impaired intestinal absorption and nutritional deficiencies (12). The non-physiological single-ventricle circulation is also associated with a mild chronic inflammatory state, similar to that observed in heart failure, which adversely affects iron homeostasis (8). The frequent use of antiplatelet and anticoagulant therapy is also of relevance.

To the best of our knowledge, only three studies addressing iron metabolism in patients with Fontan circulation have been published to date (13–15).

This study investigated the occurrence and clinical consequences of iron deficiency in adults with Fontan circulation, focusing on both hematologic aspects and exercise capacity.

## Materials and methods

In this study we enrolled adult patients with Fontan circulation followed at outpatient cardiology clinic of the University of Medical Sciences, Poznan. Information retrieved from medical records included demographic data, initial heart anatomy, prior surgical procedures, cardiac complications and the latest echocardiographic examination. We excluded patients with failing Fontan, non-sinus rhythm and severe ventricular dysfunction at routine echocardiography (ejection fraction of physiologic single ventricle <35%) to minimize cohort heterogeneity and to specifically examine exercise capacity in clinically stable Fontan physiology. None of the participants were on iron supplementation.

The study included 27 adult patients after the Fontan procedure (mean age 30.7 ± 7 years) and 26 age- and sex-matched healthy controls. The mean age at Fontan completion was 8 ± 3.7 years, and the average postoperative follow-up was 22.6 ± 6.5 years. The vast majority (92.6%) had a total cavo-pulmonary connection (TCPC), while only 7.4% had an atrio-pulmonary connection. Fenestration of the TCPC was performed in six (22.2%) patients. Single ventricle morphology was left ventricle in nineteen (70.4%) patients, right ventricle in eight (29.6%) patients. Most patients were in NYHA functional class I (63%) or II (37%). The baseline characteristics is presented in Table 1.

**Table 1:** Baseline characteristics of Fontan patients.

Clinical Records	Fontan patients (*n* = 27)
Type of congenital heart defect (%)	Tricuspid atresia 10 (37%) Double inlet left ventricle 6 (22.2%) Double outlet right ventricle 5 (18.5%) Pulmonary atresia 3 (11.1%) Mitral atresia 1 (3.7%) Hypoplastic left heart syndrome 1 (4%) CCTGA 1 (4%)
Male (%)	10 (37%)
Patient age (years)	30.7 ± 7
Age at Fontan procedure (years)	8 ± 3.7
Postoperative time (years)	22.6 ± 6.5
Type of palliation (%)	Atrio-pulmonary connection 2 (7.4%) Total cavo-pulmonary connection 25 (92.6%)
Fenestration (%)	6 (22.2%)
NYHA I/II/III/IV (%)	17 (63%)/10 (37%)/0/0
SV morphology (%)	RV: 8 (29.6%), LV: 19 (70.4%)

LV, left ventricle; NYHA, New York Heart Association; RV, right ventricle; SV, single ventricle.

### Hematological parameters

Venous blood samples were collected from participants after an overnight fast of at least 12 h. Hematological parameters, including red blood cell count (RBC), hemoglobin (HB), hematocrit (HCT), mean corpuscular volume (MCV), mean corpuscular hemoglobin (MCH), mean corpuscular hemoglobin concentration (MCHC), and red cell distribution width (RDW), were measured using an automated hematology analyzer. Serum iron, ferritin, and transferrin saturation (TSAT) were determined from fasting serum samples. Creatinine, alanine aminotransferase (ALT), were determined by standardized kinetic methods (IFCC protocol).

Iron deficiency was defined as TSAT<20% (8). Anaemia was defined as haemoglobin (Hb) <7.45 mmol/L (females) and <8.06 mmol/L (males) (16).

#### Cardiopulmonary exercise test

*Cardiopulmonary exercise treadmill test (CPET)* was performed on a treadmill using the Vyntus CPX system (Jaeger, Germany) with a ramp protocol. Patients were encouraged to exercise to volitional exhaustion, and respiratory gas exchange was measured breath-by-breath. Gas analyzers were calibrated before each test using standard reference gases. Exercise parameters were analyzed as 10-s averages and included oxygen uptake (VO_2_ and VO_2_%), resting and peak heart rate (HRrest, HRpeak), oxygen pulse (VO_2_/HR), ventilatory efficiency (VE/VCO_2_ slope), end-tidal carbon dioxide and oxygen pressures (PETCO_2_, PETO_2_), and respiratory exchange ratio (RER). Peak VO_2_ and related indices were defined as the highest 30-s averaged VO_2_ achieved during exercise. Importantly, peak VO_2_ rather than VO_2_max was reported, in line with current practice in Fontan patients, in whom achieving RER ≥1.10 is frequently not feasible. Tests were not excluded based solely on RER values. Due to mental or physical disability, cardiopulmonary exercise testing was not performed in five patients.

The Bioethical Committee of Poznan University of Medical Sciences approved the protocol—Board Review 475/19. Each participant provided informed consent to participate in this study.

### Statistics

The statistical analysis was performed using the Statistica 13 software by TIBCO. A significance level of *α* = 0.05 was adopted. The result was considered statistically significant when *p* <* α*. The normality of the distribution of variables was tested using the Shapiro–Wilk test. To compare variables between two groups, the Student's *t*-test for independent samples, the Cochran-Cox test, or the Mann–Whitney test was applied. To investigate the relationship between variables, Pearson's linear correlation coefficient (*r*) or Spearman's rank correlation coefficient (*Rs*) was calculated. To account for potential sex-related confounding, partial correlation analyses controlling for sex were performed.

## Results

### Comparison between Fontan patients and controls

Fontan patients demonstrated significantly higher RBC (4.98 ± 0.5 × 10^12^/L vs. 4.6 ± 0.4; *p* = 0.003), Hb [9.5 (7–11.1) mmol/L vs. 8.9 (7.6–10.2); *p* = 0.006], and HCT [44 (34–51)% vs. 41 (36–47); *p* = 0.01]. RDW was higher in the Fontan group [13.2 (12.4–16.3)% vs. 11.5 (10.5–14.8); *p* = 0.045], whereas TSAT was lower [24.7 (2.6–83.1)% vs. 33.5 (20.6–52.3); *p* = 0.003]. ALT [33 (18–78) U/L vs. 18.5 (8–70); *p* < 0.001], as well as NT-proBNP [241 (46–905) pg/mL vs. 52.5 (17–120); *p* < 0.001]. were markedly higher in Fontan patients, while serum creatinine was lower than in controls (69.6 ± 12.1 µmol/L vs. 79 ± 12.4; *p* = 0.007) Table 2.

**Table 2:** Clinical, laboratory and cardiopulmonary exercise test characteristics of Fontan patients and controls.

Parameter	Fontan (*n* = 27)	Controls (*n* = 26)	*p*
Male (%)	10 (37%)	10 (38.5%)	0.92
Age (years)	30.7 ± 7	32.6 ± 5.6	0.45
BMI (kg/m^2^)	22.9 ± 3.1	23.6 ± 3.6	0.54
RBC (x10^12^/L)	**4.98** **±** **0.5**	**4.6** **±** **0.4**	**0**.**003**
HB (mmol/L)	**9.5 (7–11.1)**	**8.9 (7.6–10.2)**	**0**.**006**
HCT (%)	**44 (34–51)**	**41 (36–47)**	**0**.**01**
MCV (fl)	89.1 ± 4.6	91 ± 3.5	0.1
MCH (fmol)	1.9 ± 0.1	1.9 ± 0.1	0.7
MCHC (mmol/L)	21.1 (20.3–22.5)	21 (20.9–22.8)	1
RDW (%)	**13.2 (12.4–16.3)**	**11.5 (10.5–14.8)**	**0**.**045**
Iron (umol/L)	15.4 ± 7.6	21.6 ± 6.2	0.18
Ferritin (ng/mL)	42.6 (3.9–272.5)	35 (10.1–176)	0.7
TSAT (%)	**24.7 (2.6–83.1)**	**33.5 (20.6–52.3)**	**0**.**003**
Anaemia (%)	3 (11.1%)	—	—
Creatinine (umol/L)	**69.6** **±** **12.1**	**79** **±** **12.4**	**0**.**007**
ALT (U/L)	**33 (18–78)**	**18.5 (8–70)**	**<0**.**001**
NTproBNP (pg/mL)	**241 (46–905)**	**52.5 (17–120)**	**<0**.**001**
VO_2_ (mL/kg/min)	**20.9** **±** **5.2**	**35.5** **±** **7.7**	**<0**.**001**
VO_2_%	**53.3** **±** **12**	**114.9** **±** **19.3**	**<0**.**001**
HR rest (beat/min)	87.1 **±** 16.9	79.4 **±** 13.2	0.08
HRmax (beat/min)	**139.5** **±** **25.2**	**169.7** **±** **12.7**	**<0**.**001**
VE/VCO_2_ slope	**27.8 (19.8–49.4)**	**22.4 (19–26.5)**	**0**.**001**
O_2_ pulse (mL/beat)	**9.9** **±** **2.9**	**14.9** **±** **3.5**	**<0.001**
PETCO_2_ (mmHg)	**33.9** **±** **3.8**	**40.1** **±** **5.2**	**<0.001**
PETO_2_ (mmHg)	112.9 ± 4.9	112.6 ± 6	0.85
RER	**1 (0.8–1.2)**	**1.2 (1.1–1.3)**	**<0.001**

BMI, body mass index; RBC, red blood cells; HB, hemoglobin; HCT, hematocrit; MCV, mean corpuscular volume; MCH, mean corpuscular hemoglobin; MCHC, mean corpuscular hemoglobin concentration; RDW, red cell distribution width; ALT, alanine aminotransferase; NTproBNP, N-terminal pro–B-type natriuretic peptide; VO_2_, peak oxygen uptake; HR, heart rate; VE/VCO_2_ slope, ventilation vs. carbon dioxide output slope; PETCO_2_, end-tidal carbon dioxide pressure; PETO_2_, end tidal oxygen pressure; RER, respiratory exchange ratio.

Bold values indicate statistical significance (*p* < 0.05).

Fontan patients demonstrated significantly reduced peak oxygen uptake (peakVO_2_) (20.9 ± 5.2 mL/kg/min vs. 35.5 ± 7.7; *p* < 0.001), as well as percent predicted VO_2_ (53.3 ± 12% vs. 114.9 ± 19.3; *p* < 0.001) when compared to controls. In comparison to controls Fontan group had significantly lower HRmax (139.5 ± 25.2 bpm vs. 169.7 ± 12.7; *p* < 0.001), while VE/VCO_2_ slope was higher [27.8 (19.8–49.4) vs. 22.4 (19–26.5); *p* = 0.001]. The RER was significantly lower in the Fontan group than in the controls [1.0 (0.8–1.2) vs. 1.2 (1.1–1.3), *p* < 0.001], indicating that a proportion of tests may have been submaximal. Accordingly, peak VO_2_ rather than VO_2_max was used for analyses Table 2.

### Comparison according to iron status (TSAT <20% vs. ≥20%)

Among the Fontan group, seven patients (26%) had low TSAT (<20%). This subgroup consisted entirely of women and presented with lower RBC (4.6 ± 0.5 vs. 5.1 ± 0.4 × 10^12^/L; *p* = 0.02), Hb [7.9 (7–9.6) vs. 9.9 (9.1–11.1) mmol/L; *p* = 0.002], and HCT (39.7 ± 5.3% vs. 46.1 ± 2.9; *p* < 0.001) when compared to patients without iron deficiency. Red cell indices were smaller in the low-TSAT group: MCV (85.4 ± 4.9 fL vs. 90.5 ± 3.8; *p* = 0.01), MCH (1.8 ± 0.1 fmol vs. 1.9 ± 0.1; *p* = 0.001), and MCHC (20.7 ± 0.3 mmol/L vs. 21.5 ± 0.6; *p* = 0.002), whereas RDW was higher [13.5 (12.9–16.3) vs. 13 (12.4–15); *p* = 0.017] when compared to patients with TSAT ≥20%.

As expected, iron (7 ± 4 vs. 18.3 ± 6.3 µmol/L; *p* < 0.001), ferritin [7.8 (3.9–56.1) vs. 74.2 (4.3–272.5) ng/mL; *p* = 0.004], and TSAT [7.6 (2.6–18.1) vs. 27 (20–83.1)%; *p* < 0.001] were markedly reduced in the iron-deficient subgroup.

Regarding metabolic and functional parameters, patients with TSAT <20% had significantly lower serum creatinine (61.8 ± 7.8 µmol/L vs. 72.3 ± 12.3; *p* = 0.04) and ALT [26 (18–32) U/L vs. 36.5 (22–78); *p* = 0.002].

When compared to patients with TSAT ≥20%, exercise performance was impaired in the iron-deficient group: peak VO_2_ was lower (17.6 ± 5.1 vs. 22.1 ± 4.9 mL/kg/min, *p* = 0.04), and VE/VCO_2_ slope was higher (33.6 ± 8.3 vs. 27.0 ± 4.0, *p* = 0.021). PETCO_2_ was also lower (30.7 ± 3.9 mmHg vs. 35 ± 3.2; *p* = 0.015). Other parameters, including HRrest, HRmax, O_2_ pulse, and RER, showed no significant differences between the groups Table 3.

**Table 3:** Clinical, laboratory and cardiopulmonary exercise test characteristics in Fontan patients with regard to iron deficiency.

Parameter	TSAT <20% (*n* = 7)	TSAT ≥20% (*n* = 20)	*p*
Sex (%)			**0** **.** **02**
Female	7 (100%)	10 (50%)	
Male	0	10 (50%)	
Age (years)	32 ± 7.3	30.3 ± 7	0.6
BMI (kg/m^2^)	22.2 ± 1.5	23.2 ± 3.5	0.47
RBC (x10^12^/L)	**4.6** ± **0.5**	**5.1** ± **0.4**	**0**.**02**
HB (mmol/L)	**7.9 (7–9.6)**	**9.9 (9.1–11.1)**	**0**.**002**
HCT (%)	**39.7** ± **5.3**	**46.1** ± **2.9**	**<0**.**001**
MCV (fl)	**85.4** ± **4.9**	**90.5** ± **3.8**	**0**.**01**
MCH (fmol)	**1.8** ± **0.1**	**1.9** ± **0.1**	**0**.**001**
MCHC (mmol/L)	**20.7** ± **0.3**	**21.5** ± **0.6**	**0**.**002**
RDW (%)	**13.5 (12.9–16.3)**	**13 (12.4–15)**	**0**.**017**
Iron (umol/L)	**7** ± **4**	**18.3** ± **6.3**	**<0**.**001**
Ferritin (ng/mL)	**7.8 (3.9–56.1)**	**74.2 (4.3–272.5)**	**0**.**004**
TSAT (%)	**7.6 (2.6–18.1)**	**27 (20–83.1)**	**<0**.**001**
Anaemia %	3 (42.9%)	—	—
Creatinine (umol/L)	**61.8** ± **7.8**	**72.3** ± **12.3**	**0**.**04**
ALT (U/L)	**26 (18–32)**	**36.5 (22–78)**	**0**.**002**
NTproBNP (pg/mL)	266 (56–691)	221 (46–905)	0.8
VO_2_ (mL/kg/min)	**17.6** ± **5.1**	**22.1** ± **4.9**	**0**.**04**
VO2%	47.3 ± 12.9	55.6 ± 11.2	0.2
HR rest (beat/min)	82.5 ± 20.5	88.9 ± 15.8	0.4
HRmax (beat/min)	138.2 ± 23.2	140 ± 26.6	0.9
VE/VCO_2_ slope	**33.6** ± **8.3**	**27** ± **4**	**0**.**021**
O_2_ pulse (mL/beat)	8.3 ± 2.6	10.6 ± 2.8	0.09
PETCO_2_ (mmHg)	**30.7** ± **3.9**	**35** ± **3.2**	**0**.**015**
PETO_2_ (mmHg)	114.5 ± 4.2	112.4 ± 5.2	0.4
RER	0.99 ± 0.13	1 ± 0.04	0.82

BMI, body mass index; RBC, red blood cells; HB, hemoglobin; HCT, hematocrit; MCV, mean corpuscular volume; MCH, mean corpuscular hemoglobin; MCHC, mean corpuscular hemoglobin concentration; RDW, red cell distribution width; ALT, alanine aminotransferase; NTproBNP, N-terminal pro–B-type natriuretic peptide; VO_2_, peak oxygen uptake; HR, heart rate; VE/VCO_2_ slope, ventilation vs. carbon dioxide output slope; PETCO_2_, end-tidal carbon dioxide pressure; PETO_2_, end tidal oxygen pressure; RER, respiratory exchange ratio.

Bold values indicate statistical significance (*p* < 0.05).

### Correlation analysis

In the Fontan group, several associations were observed between hematologic, metabolic, and functional variables. TSAT correlated positively with HB (*r* = 0.55, *p* = 0.003), HCT (*r* = 0.49, *p* = 0.008), MCHC (*r* = 0.49, *p* = 0.008), serum iron (*r* = 0.69, *p* < 0.001), and ferritin (*r* = 0.52, *p* = 0.005). TSAT also showed positive correlations with peakVO_2_ (*r* = 0.51, *p* = 0.015) and PETCO_2_ (*r* = 0.49, *p* = 0.02), and a negative correlation with VE/VCO_2_ slope (*r* = –0.55, *p* = 0.009). Partial correlation analyses controlling for sex were performed. After adjustment, transferrin saturation showed a trend toward a positive association with peak VO_2_ (partial *r* = 0.43, *p* = 0.06) and was inversely associated with VE/VCO_2_ slope (partial *r* = −0.49, *p* = 0.027).

Peak VO_2_ correlated positively with HCT (*r* = 0.49, *p* = 0.02), ALT (*r* = 0.47, *p* = 0.02) and oxygen pulse (*r* = 0.47, *p* = 0.03), and negatively with VE/VCO_2_ slope (*r* = –0.51, *p* = 0.02).

RDW correlated negatively with HCT (*r* = −0.6, *p* < 0.001), MCV (*r* = –0.47, *p* = 0.01), MCH (*r* = –0.52, *p* = 0.005), and MCHC (*r* = –0.39, *p* = 0.04). RDW also correlated negatively with ferritin (*r* = –0.54, *p* = 0.004) and TSAT (*r* = –0.56, *p* = 0.002). Functionally, RDW correlated negatively with O_2_ pulse (*r* = –0.42, *p* = 0.048) Table 4.

**Table 4:** Correlations of VO_2_, PETCO_2_, VE/VCO_2_, TSAT, RDW.

Parameter	VO_2_	PETCO_2_	VE/VCO_2_	TSAT	RDW
Sex (%)	ns	ns	ns	ns	ns
Age (years)	ns	ns	ns	ns	ns
BMI (kg/m^2^)	ns	ns	ns	ns	ns
RBC (x10^12^/L)	ns	ns	ns	0.45 **0.02**	(−0.39) **0.04**
HB (mmol/L)	ns	ns	ns	0.55 **0.003**	(−0.65) **<0.001**
HCT (%)	0.49 **0.02**	ns	ns	0.49 **0.008**	(−0.6) **<0.001**
MCV (fl)	ns	ns	ns	ns	(−0.47) **0.01**
MCH (fmol)	ns	ns	ns	0.38 **0.049**	(−0.52) **0.005**
MCHC (mmol/L)	ns	ns	ns	0.49 **0.008**	(−0.39) **0.04**
RDW (%)	ns	ns	ns	ns	—
Iron (umol/L)	ns	ns	ns	0.69 **<0.001**	ns
Ferritin (ng/mL)	ns	ns	ns	0.52 **0.005**	(−0.54) **0.004**
TSAT (%)	0.51 **0.015**	0.49 **0.02**	(−0.55) **0.009**	—	(−0.56) **0.002**
Anaemia %	ns	ns	ns	ns	ns
Creatinine (umol/L)	ns	ns	ns	0.48 **0.009**	(−0.47) **0.01**
ALT (U/L)	0.47 **0.02**	0.57 **0.005**	(−0.48) **0.03**	ns	ns
NTproBNP (pg/mL)	ns	ns	ns	ns	ns
VO_2_ (mL/kg/min)	—	ns	(−0.51) **0.02**	0.51 **0.015**	ns
VO_2_%	—	ns	ns	ns	ns
HR rest (beat/min)	ns	ns	ns	ns	ns
HRmax (beat/min)	ns	ns	ns	ns	ns
VE/VCO_2_ slope	(−0.51) **0.02**	(−0.7) **<0.001**	ns	(−0.55) **0.009**	ns
O_2_ pulse (mL/beat)	0.47 **0.03**	ns	(−0.54) **0.01**	ns	(−0.42) **0.048**
PETCO_2_ (mmHg)	ns	ns	(−0.7) **<0.001**	0.49 **0.02**	ns
PETO_2_ (mmHg)	ns	(−0.58) **0.005**	ns	ns	ns
RER	ns	ns	ns	ns	ns

BMI, body mass index; RBC, red blood cells; HB, hemoglobin; HCT, hematocrit; MCV, mean corpuscular volume; MCH, mean corpuscular hemoglobin; MCHC, mean corpuscular hemoglobin concentration; RDW, red cell distribution width; ALT, alanine aminotransferase; NTproBNP, N-terminal pro–B-type natriuretic peptide; VO_2_, peak oxygen uptake; HR, heart rate; VE/VCO_2_ slope, ventilation vs. carbon dioxide output slope; PETCO_2_, end-tidal carbon dioxide pressure; PETO_2_, end tidal oxygen pressure; RER, respiratory exchange ratio.

Bold values indicate statistical significance (*p* < 0.05).

## Discussion

Our findings complement recent observations from larger Fontan cohorts demonstrating that iron deficiency is common in this population and is associated with impaired exercise capacity, even in the absence of overt anemia (13–15). In our group of adult patients after the Fontan procedure, nearly one quarter (21%) exhibited ID. This proportion is lower than that reported by Nakashima et al. (15), who identified ID in 73.4% of 94 patients, yet higher than the rate observed by Tomkiewicz-Pajak et al. (14), where only 12.5% of the studied group was affected. It should be noted that the latter study employed a somewhat different methodology for assessing iron deficiency (14). In our cohort, as in the other cited studies, women predominated among patients with ID, a finding most likely attributable to menstrual blood loss in these typically young female patients. Interestingly, all of these observed rates of ID are markedly lower than those reported in the general heart failure population (33%–74%) (4, 5). This discrepancy likely reflects the older age and substantially greater burden of comorbid conditions in the overall heart failure cohort compared with the younger and less comorbid Fontan population.

The primary factor contributing to ID in patients with Fontan circulation is the resulting elevation of systemic venous pressure. Passive hepatic congestion leads to increased production of hepcidin, a peptide that reduces intestinal iron absorption and inhibits the release of stored iron from tissues, thereby contributing to so-called functional ID (17, 18). Hepcidin synthesis in the liver is further enhanced by the chronic inflammatory state characteristic of Fontan circulation, driven by activation of the pro-inflammatory cytokine cascade typically observed in heart failure (IL-6, IL-1, TNF-α) (19). This process also stimulates the production of ferritin, a key protein involved in iron storage (8, 19). Disturbances in intestinal absorption caused by venous congestion, which may contribute to malnutrition, are another important factor influencing iron homeostasis in our patients (19, 20). The most extreme manifestation of this condition is protein-losing enteropathy, in which ID has already been demonstrated by Polish investigators (14).

As it is well established in the pathophysiology of iron redistribution, anaemia emerges only at a later stage of tissue iron depletion (21, 22). This likely explains why van Hassel et al. (13) identified anaemia in only 9% of their group, and why, in our study, it was present in 11% of patients—exclusively among those with confirmed ID. At the same time, fewer than half of the patients with ID exhibited anaemia. This phenomenon may be explained by increased hepcidin concentrations in these patients, which impair the mobilization of iron from maturing erythroblasts and thereby help maintain erythropoiesis at a relatively stable level despite underlying ID (17, 18). Anaemia was not documented in the study conducted by Tomkiewicz-Pajak et al. (14). Moreover, these authors, similar to our findings, observed features of erythrocytosis in patients with Fontan circulation. This phenomenon can be explained by a compensatory response to chronic hypoxemia, resulting from persistent veno-venous collaterals or a patent fenestration. In our group, nearly one quarter of patients had a patent fenestration. These findings are consistent with observations reported by other authors (23, 24). Additionally, patients after the Fontan procedure often exhibit reduced plasma volume and a tendency toward hemoconcentration (24). This is considered part of the adaptive response to the abnormal hemodynamics and low venous flow characteristic of this population. As a result, the increased red cell mass may enhance oxygen transport, but it simultaneously raises blood viscosity and the risk of thromboembolic complications (25).

Our analysis confirmed a significant impairment of exercise capacity in patients after the Fontan procedure, consistent with numerous previous studies, whose results showed only minor variation depending on the age and sex distribution of the studied groups (26–28). The Fontan procedure is, by nature, a palliative intervention. Cardiac output depends on the ability of the single ventricle to impart sufficient kinetic energy to the blood so that, despite gravity and in the absence of a subpulmonary ventricle, it can be propelled into the pulmonary circulation (2). Sufficiently low pulmonary vascular resistance, in turn, allows the oxygenated blood to return and ensures adequate filling of the systemic ventricle—that is, its preload (1). To sustain adequate exercise capacity, the fragile hemodynamic balance of the Fontan circulation—often described as functioning in a “parallel” configuration—relies heavily on metabolic processes that modulate its performance, including iron homeostasis. As it is well established, ferrum plays a key role in maintaining oxygen homeostasis: it is essential for oxygen transport (hemoglobin), its storage in muscle tissue (myoglobin), and mitochondrial oxidative–energetic processes (29–31). ID leads to disturbances in skeletal muscle metabolism, impaired regeneration, and reduced exercise capacity—effects that have been well demonstrated in the general population (29, 32). In line with recent data from adult Fontan populations, we observed that iron status was closely related to peak VO_2_ and ventilatory efficiency (13, 15). We observed a significant correlation between TSAT and oxygen uptake during cardiopulmonary exercise (peak VO_2_), a finding consistent with the study conducted by van Hassel et al. and Nakashima et al. (13, 15). Our observations extend these findings by suggesting that ID may also be linked to altered ventilatory responses during exercise, as reflected by higher VE/VCO_2_ slope and lower PETCO_2_. These findings indicate that ID may be associated not only with impaired oxygen transport but also with altered ventilatory efficiency, potentially reflecting limitations in respiratory muscle performance and metabolic capacity. In this context, inefficient ventilation may be related to impaired mitochondrial oxidative metabolism, leading to an earlier shift toward anaerobic pathways, increased CO_2_ production, and an exaggerated ventilatory response (29, 33).

In line with the observations by van Hassel et al., the majority of our patients did not achieve maximal effort during cardiopulmonary exercise testing, as defined by reaching an RER ≥1.1 (13). Moreover, those authors reported that patients with ID were more likely to perform submaximal exercise tests. Exercise intolerance in individuals with a Fontan circulation is multifactorial; in addition to the limited ability of the single ventricle to augment cardiac output in response to increased metabolic demand, other factors such as sedentary lifestyle, obesity, and aging also contribute to reduced exercise performance (34). Therefore, regular physical activity has been associated with a positive exercise capacity trajectory and improvements in oxygen pulse and VO_2_ at the anaerobic threshold in patients with a Fontan circulation, suggesting a substantial contribution of peripheral and preload-related mechanisms (35).

To address potential sex-related confounding, we performed partial correlation analyses controlling for sex. After adjustment, transferrin saturation remained inversely associated with VE/VCO_2_ slope and showed a trend toward a positive association with peak VO_2_. These findings suggest that the observed relationships between iron status and cardiopulmonary exercise performance are not solely explained by sex differences.

An indirect indication of the relevance of iron homeostasis to the pathophysiology of impaired exercise capacity in this group is the reduced creatinine concentration, which reflects diminished muscle mass and underlying malnutrition (20). Reduced muscle mass lowers both energy reserves and respiratory muscle strength, which directly contributes to diminished exercise tolerance. At the same time, malnutrition, commonly observed in patients with Fontan circulation, may further exacerbate ID through reduced intake and impaired absorption of iron, creating a vicious cycle of malnutrition, muscle loss, and diminished aerobic capacity (36).

## Conclusions

Some patients after the Fontan procedure exhibit a significant iron deficiency resulting from the hemodynamic consequences of this palliative circulation. This deficiency adversely affects their exercise capacity, underscoring the need for active monitoring of iron status parameters in this population.

### Limitations of the study

The limitations of our study include the small sample size and the heterogeneity of the cohort, stemming from the underlying diversity of congenital heart defects, which reduces statistical power and limits the generalizability of our findings. The markedly higher proportion of women in our sample represents another limitation. Similar to other CPET studies in Fontan patients, a substantial proportion of tests in our cohort did not reach conventional maximal effort criteria, reinforcing the use of peak VO_2_ rather than VO_2_max in this population. Despite these constraints, our results provide important hypotheses and highlight areas that warrant further investigation.

## Data Availability

The original contributions presented in the study are included in the article/Supplementary Material, further inquiries can be directed to the corresponding author.
